# Supplementary material for: Development and Validation of a Mobile Phone Application Developed for Measuring Dietary Fiber Intake
Source: Nutrients. 2021 Jun 22;13(7):2133. doi: 10.3390/nu13072133 (PMC8308258; doi:10.3390/nu13072133)
Supplement: Supplementary file 1 [file nutrients-13-02133-s001.zip › nutrients-1255492-supplementary.pdf]

Supplementary materials

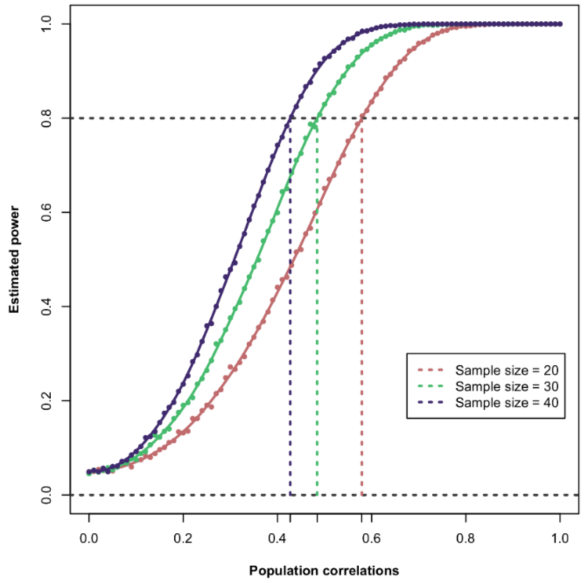

Figure S1. Power calculation with simulated functions for population correlations for three sample sizes.

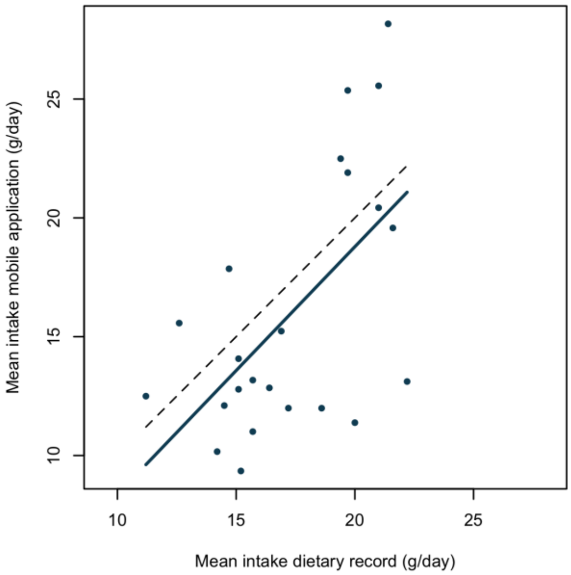

Figure S2. Correlation plot of the coherence between fiber intake (g) measured by the mobile phone application and the dietary record (n = 23). Outliers are excluded. The dashed line presents a unity line (x = y).

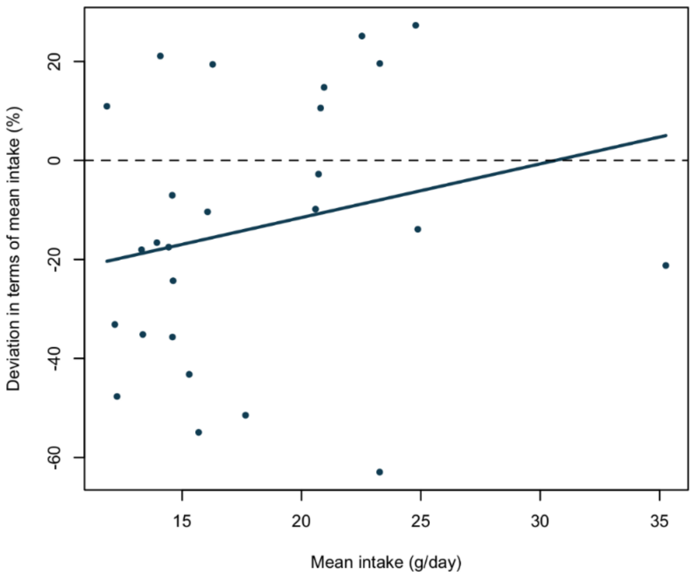

Figure S3. Regression analysis including the deviation in terms of percentage of mean fiber intake as measured by the mobile phone application and the dietary record, and the mean fiber intake of the methods (n = 26). The solid line represents the mean relative deviation. The dashed line at zero is included for reference.

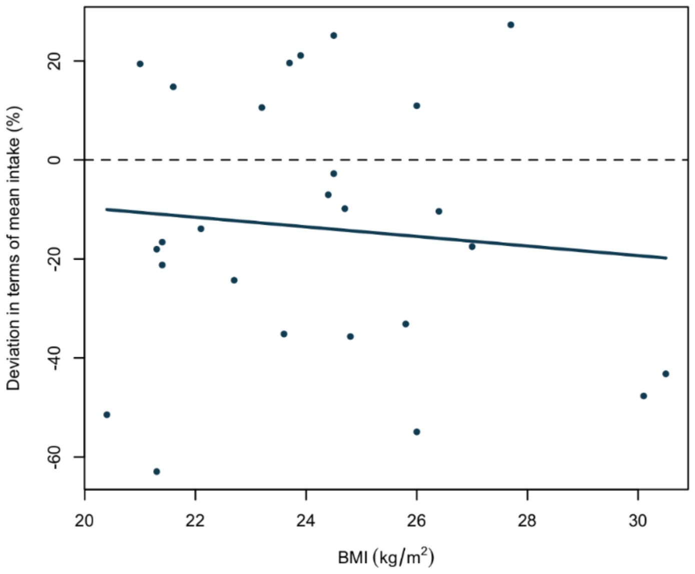

Figure S4. Regression analysis including the deviation in terms of percentage of mean fiber intake as measured by the mobile phone application and the dietary record and BMI (n = 26). The solid line represents the mean relative deviation. The dashed line at zero is included for reference.

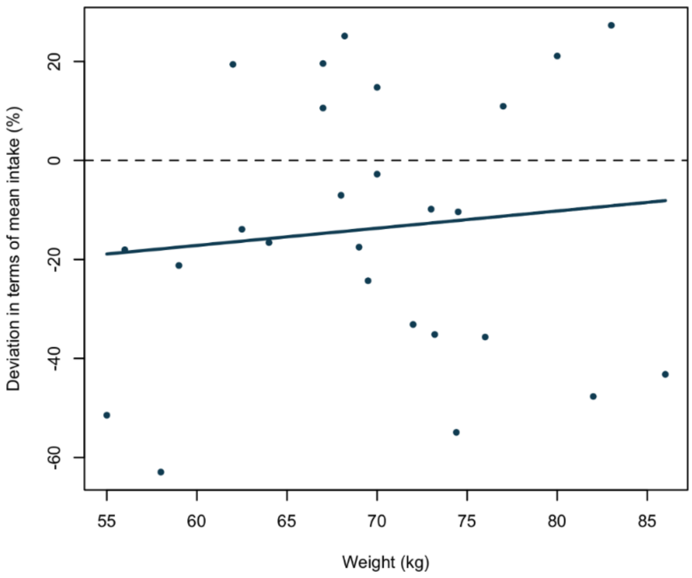

Figure S5. Regression analysis including the deviation in terms of percentage of mean fiber intake as measured by the mobile phone application and the dietary record and weight (n = 26). The solid line represents the mean relative deviation. The dashed line at zero is included for reference.
